# Comparison of Manual Wheelchair and Pushrim-Activated Power-Assisted Wheelchair Propulsion Characteristics during Common Over-Ground Maneuvers

**DOI:** 10.3390/s21217008

**Published:** 2021-10-22

**Authors:** Mahsa Khalili, Garrett Kryt, W. Ben Mortenson, Hendrik F. Machiel Van der Loos, Jaimie Borisoff

**Affiliations:** 1 School of Biomedical Engineering, University of British Columbia, 2222 Health Sciences Mall, Vancouver, BC V6T 1Z3, Canada; vdl@mech.ubc.ca; 2 Centre for Applied Research and Innovation, British Columbia Institute of Technology, 3700 Willingdon Avenue, Burnaby, BC V5G 3H2, Canada; gkryt@bcit.ca (G.K.); Jaimie_Borisoff@bcit.ca (J.B.); 3 Department of Occupational Science and Occupational Therapy, University of British Columbia, 2211 Wesbrook Mall, Vancouver, BC V6T 2B5, Canada; ben.mortenson@ubc.ca; 4 International Collaboration on Repair Discoveries, Blusson Spinal Cord Centre, 818 West 10th Avenue, Vancouver, BC V5Z 1M9, Canada; 5 Rehabilitation Research Program, G.F. Strong Rehabilitation Centre, 4255 Laurel Street, Vancouver, BC V5Z 2G9, Canada; 6 Department of Mechanical Engineering, University of British Columbia, 6250 Applied Science Lane, Vancouver, BC V6T 1Z4, Canada

**Keywords:** manual wheelchair, pushrim-activated power-assisted wheel, kinetics of propulsion, kinematics of motion

## Abstract

Pushrim-activated power-assisted wheels (PAPAWs) are assistive technologies that use force sensor data to provide on-demand propulsion assistance to manual wheelchair users. However, available data about kinetic and kinematic of PAPAW use are mainly limited to experiments performed on a treadmill or using a dynamometer. In this work, we performed experiments to gather kinetics of wheelchair propulsion and kinematics of wheelchair motion for a variety of over-ground wheelchair maneuvers with a manual wheelchair with and without PAPAWs. Our findings revealed that using PAPAWs can significantly reduce the propulsion effort and push frequency. Both linear and angular velocities of the wheelchair were significantly increased when using PAPAWs. Less force and push frequency could potentially reduce risk of chronic upper limb injury. Higher linear velocity could be desirable for various daily life activities; however; the increase in the angular velocity could lead to unintended deviations from a desired path. Future research could investigate PAPAW controllers that amplify the desired intentions of users while mitigating any unwanted behaviours.

## 1. Introduction

Manual wheelchairs (MWCs) are the most commonly prescribed wheeled mobility assistive devices (WMADs) for people with ambulatory limitations [1]. In 2012, it was estimated that about 68% of WMAD users in Canada relied on MWCs [2]. MWCs are relatively lightweight, compact, and easy to maneuver [3]. In addition, MWCs have the potential to enhance physical activity [4,5]. Despite these positive aspects, there are several disadvantages associated with short- and long-term use of MWCs. For instance, wheeling a MWC is a physically demanding task, and over time use of these devices can increase the risk of secondary health conditions such as upper extremity joint pain or repetitive strain injuries [1,6,7].

Various powered/non-powered “add-on” components have been developed which can mitigate or eliminate the physical load of MWC use [8]. Some examples of these add-ons include powered wheels, front-mounted attachments, and rear-mounted attachments [9]. The findings of a previous research study investigating perceptions of powered add-ons revealed that powered wheels were perceived to be the most effective attachments for indoor and outdoor mobility, as they look and feel most similar to a MWC [10]. Powered wheels replace the conventional wheels of a MWC and can be controlled either by a joystick or direct pushrim interactions. Joystick-driven powered wheels, such as e-fixTM [11], convert a MWC to a compact powered system and enable users to easily navigate the wheelchair with a joystick. Pushrim-activated power-assisted wheels (PAPAWs) are another type of powered wheels with built-in sensors to detect the user input force/torque to the pushrims. The force/torque measurements are fed back to PAPAW controllers and are used to determine the level of propulsion assistance provided to the user [12]. Therefore, PAPAW users can propel a wheelchair in the same way as propelling a MWC while exerting less effort [13]. Examples of PAPAWs include the Alber e-motion^®^ [14] and Yamaha NAVIONE [15].

The functional, physical, and physiological performance of MWC and PAPAW users has been examined previously. There is strong evidence in the literature supporting the use of PAPAWs. For instance, wheelchair users were found to have lower heart rates [13,16,17,18], lower oxygen consumption [17,18,19], and higher mechanical efficiency [19] when using PAPAWs compared with MWCs. A comparison between the kinetic characteristics of MWC and PAPAW propulsion on a treadmill moving at a constant velocity revealed that the user input (peak) force to the pushrims was significantly lower when using PAPAWs compared with manual wheels [20,21]. PAPAWs can also improve the ability to navigate challenging terrains such as ramps or inclines [16,22,23]. In addition to various physical and functional benefits [16,24], PAPAWs have the potential to enhance users’ independence and promote participation [10,25].

Mixed findings were reported regarding the effects of PAPAW use on stroke frequency. For instance, the findings of one study involving 15 skilled wheelchair users revealed a significant reduction in the stroke frequency when propelling PAPAWs on a dynamometer (at different resistance levels) compared with MWC propulsion [17]. However, another study reported a significant increase in the stroke frequency when comparing characteristics of MWC and PAPAW propulsion on a treadmill with a fixed speed [21]. A third study, involving MWC and PAPAW propulsion tests on a dynamometer with two different speeds and various resistance levels, found no significant change in the stroke frequency [26]. Although PAPAW users have described benefiting from the additional assist torque when performing high-intensity activities, they described having difficulty performing tasks that required fine control over the wheelchair motion (e.g., wheelies or turns) [27]. Since available data supporting the benefits of PAPAW use are mainly limited to experiments performed on a treadmill or dynamometer, further investigations were recommended to examine the biomechanics of wheelchair propulsion and the efficacy of PAPAW use in more realistic test settings (e.g., including obstacle courses) [28,29].

The main objective of this study was to examine and compare the performance and characteristics of a MWC and PAPAWs when performing common daily life wheelchair maneuvers. In addition, we sought to measure participants’ perceptions regarding the workload of wheelchair propulsion when using powered and unpowered wheels. 

## 2. Methods

### 2.1. Hardware Setup and Participants

**Figure 1:**
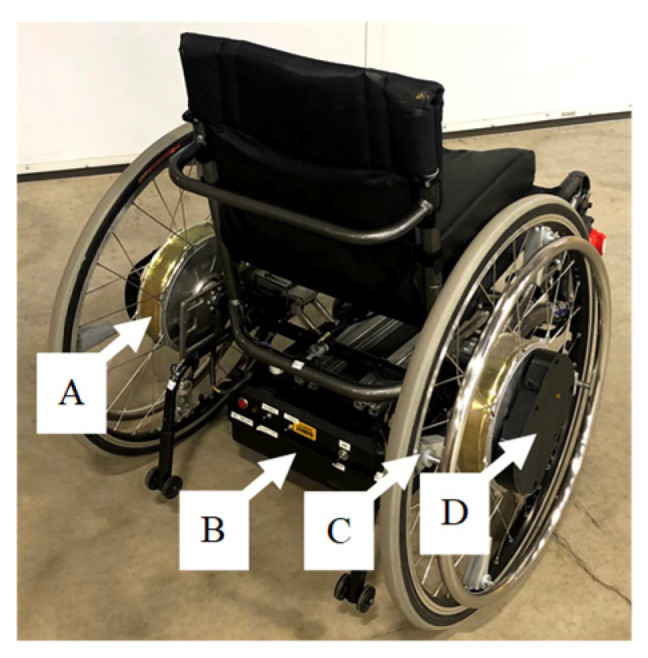
BCIT Wheelchair. A: In-hub Motor; B: Microcontroller, motor controller, IMU (frame), batteries; C: Force sensor; D: Microcontroller, IMU (wheels), batteries.

The PAPAWs that were used in this study were developed at the British Columbia Institute of Technology (BCIT). The test setup consisted of two PAPAWs mounted to an Elevation^TM^ manual wheelchair (PDG Mobility, Vancouver, BC, Canada; Figure 1). Detailed information about the mechanical and electrical specifications of the BCIT wheelchair are listed in Table 1 and Table 2. 

Study participants included one skilled MWC user and three able-bodied individuals who had prior experience with MWC and PAPAW use. The demographics of all study participants are presented in Table 3. Each participant was provided with information about the safety and intent of the tests, and participants gave their informed consent before participating in the experiments. This study was approved by the Behavioural Research Ethics Board of the University of British Columbia.

### 2.2. Experimental Protocol

Two experiments were conducted on two separate days: (1) MWC tests and (2) PAPAW tests. All tests were performed on a smooth level concrete in the MAKE+ lab located at BCIT’s Centre for Applied Research and Innovation (Vancouver, BC). All tests were completed in less than one hour. Detailed information about these experiments is presented in the remainder of this section.

**Figure 2:**
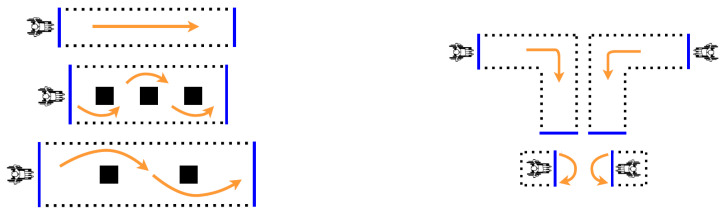
Wheelchair maneuvers—(**Left**) (top to bottom): (1) straight forward; (2) maneuvering around obstacles placed 1.5 m apart; (3) maneuvering around obstacles placed 3.5 m apart; (**Right**) (top to bottom): (4, 5) straight forward with a 90° left/right turn; (6, 7) turning 180° left/right in place. Each maneuver was repeated 12 times.

**MWC Tests:** Study participants were instructed to perform seven pre-defined wheelchair maneuvers (Figure 2). The order of these maneuvers was randomized for each participant. The complete list of these maneuvers and a detailed description of each are presented in Table 4. Start and stop points as well as the direction of motion for different wheelchair activities were marked on the floor. Participants were instructed to start each maneuver from rest, follow the predefined path at a self-selected speed, and stop at a predefined location. All maneuvers were repeated 12 times (a total of 84 tests for each participant). 

The abovementioned maneuvers were performed with the BCIT wheelchair while no power assistance was provided by the wheels (i.e., MWC propulsion). Participants were given the opportunity to familiarize themselves with the wheelchair and maneuvers before the start of the experiment, and they could take a break between different tests if requested. One researcher was responsible for instructing the participants to start the trial as well as initiating/terminating the data acquisition process. The experiments were recorded by a second researcher. 

Kinematics of wheelchair motion were obtained using three 9-axis IMU (BNO055, Bosch Sensortec, GmbH, Germany) with a triaxial gyroscope and accelerometer. One IMU was mounted on the wheelchair frame and one IMU module was attached to each wheel (Figure 1). Kinetics of wheelchair propulsion (i.e., user input torque to the pushrims) were measured using a force-calibrated Hall effect sensor. The Hall sensor produced a voltage proportional to the change in position of a link that was attached to the wheelchair pushrim. The link was centered in a housing that included elastomers that controlled the return-to-center of the Hall sensor. When the user imparted a force on the pushrim, the sensor link moved a proportional amount based on the stiffness of the elastomer and returned to center when the input force was removed. Further electronic specifications are listed in Table 2. The wheel modules recorded and streamed the IMU and Hall sensor data via a Bluetooth serial connection to the frame module microcontroller. The frame module mapped the Hall sensor data to a calibrated input force. All measurements were sampled at 20 Hz and logged on an onboard SD card. 

**Figure 3:**
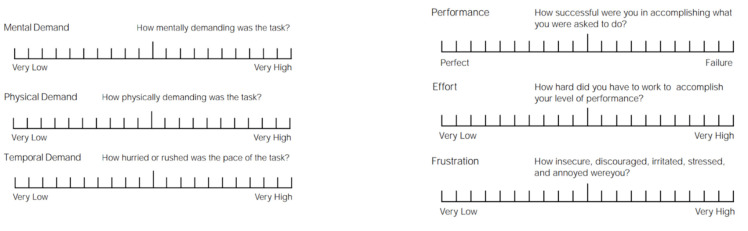
The NASA-TLX questionnaire.

The NASA Task Load Index (NASA-TLX) questionnaire [30] was used to assess task-load demand (i.e., mental, physical, temporal, performance, effort, and frustration) of the MWC tests (Figure 3). The NASA-TLX scale ranges from very low (0) to very high (100), representing the level of ease or difficulty for each domain (lower scores are more favourable). 

**PAPAW Tests:** On day 2, similar experimental protocols were followed as day one (i.e., same wheelchair maneuvers, same kinetic/kinematic measurements). However, all experiments were performed using the BCIT PAPAW, meaning that the in-hub motors were providing propulsion assistance to participants. In this case, data from the left and right wheel force sensors were used to determine the left and right wheel motor commands, respectively. A proportional motor signal based on the user input force was then output to the motor controller. The motor controller operated in closed loop torque-control mode, where the motor command controls the current regardless of motor speed. Similar to MWC tests, all measurements were sampled at 20 Hz and logged on an onboard SD card. Future output to the motor controller may include prior machine learning algorithms and/or other signal processing on the microcontroller to improve and/or customize the PAPAW control. The NASA-TLX questionnaire was administered upon the completion of these wheelchair tests. 

### 2.3. Data Analysis

Five kinetic and kinematic features were selected to compare the performance of MWC and PAPAW tests that are (1, 2) the user pushrim torque to the left and right wheels; (3, 4) linear and angular velocity of the wheelchair (angular velocities of the left and right wheels were used to calculate the linear and angular velocity of the wheelchair for different maneuvers); and (5) the number of strokes (i.e., push frequency). The maximum (max) value and root mean square (RMS) of the torque and velocity features were calculated for all wheelchair maneuvers. A total of 400 data points (4 participants, 5 maneuvers (data from the 180 turns were dropped since they were notably different from the rest of the measurements), 10 trials (data from the first two trials were dropped for all maneuvers to account for potential learning effects and measurements of 10 trials were analyzed), and 2 wheel types) were analyzed. Descriptive statistics (mean, standard deviation, median, range) were used to summarize the results. All variables violated the Shapiro–Wilk test of normality (*p*-value ≤ 0.05); therefore, the Wilcoxon signed-rank tests were used for pairwise comparisons (For this comparison, we assumed that kinematic and kinetic characteristics of MWC and PAPAW tests are paired (e.g., data from “trial 10” of “straight forward” of MWC tests were compared with “trial 10” of “straight forward” PAPAW tests).). The significance level was adjusted using the Bonferroni correction based on 10 pairwise comparisons (*p*-value ≤ 0.005). 

Radar graphs were used to visualize the NASA-TLX scores of all tests. Scores of the MWC and PAPAW tests were compared using the Wilcoxon signed-rank tests (*p*-value ≤ 0.05). Statistical analysis for this study was performed using SPSS [31]. 

## 3. Results

**Figure 4:**
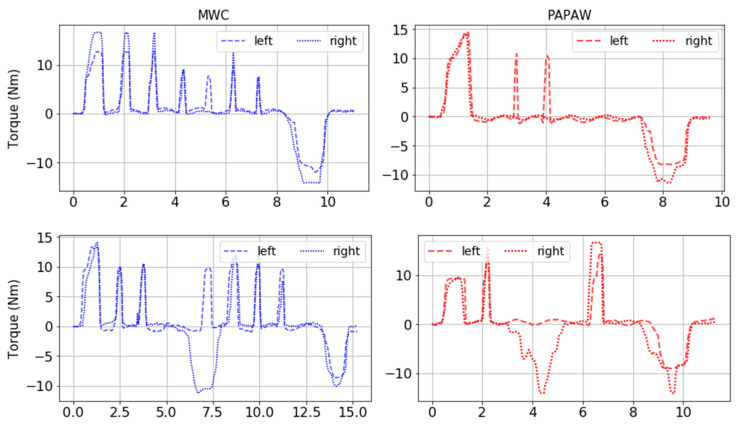

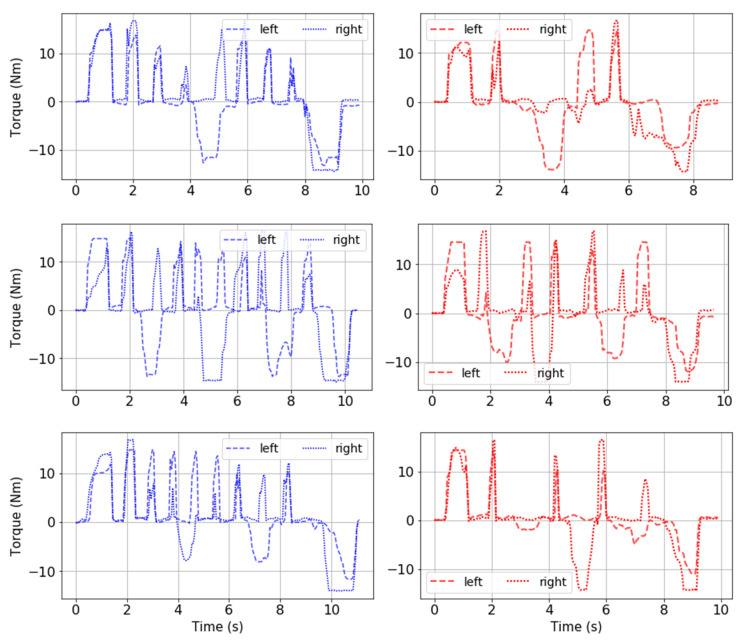
Kinetic characteristics of a subset of MWC tests (**left**) and PAPAW tests (**right**) for Participant 3. Wheelchair maneuvers from top to bottom: “Straight forward”, “Turn 90° right”, “Turn 90° left”, “Avoid obstacles 1.5 m”, “Avoid obstacles 3.5 m”.

Kinetic characteristics of a representative subset of MWC and PAPAW tests for Participant 3 (i.e., skilled wheelchair user) are shown in Figure 4. The cyclic pattern of the propulsive torque to the left and right wheels represents the push and recovery phases during wheelchair motion. The propulsive and braking torques that are synchronously applied to opposite wheels indicate a turning phase. A comparison of MWC and PAPAW data shown in these graphs suggests that, for all maneuvers, the task completion time and push frequency were lower when using PAPAWs. 

**Figure 5:**
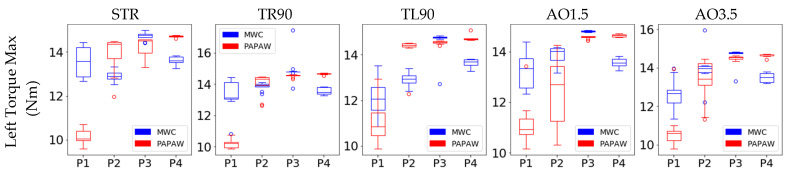

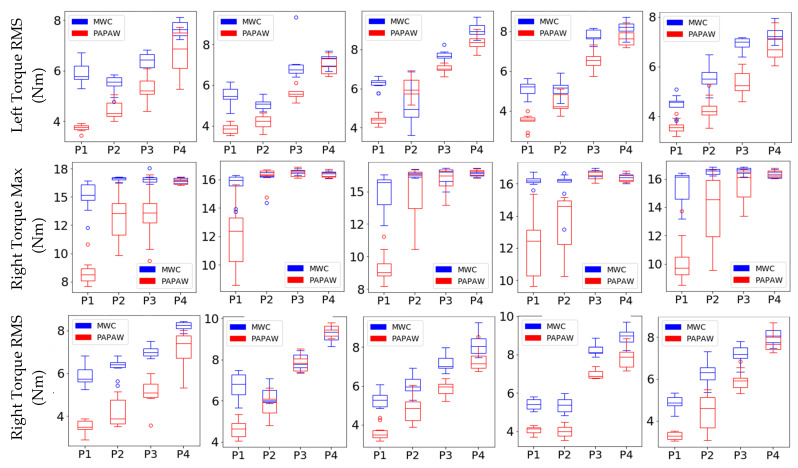
Summary of kinetic characteristics for all wheelchair maneuvers. Each box extends from the Q1 to Q3 quartile values of the data, with a line at the median (Q2). The whiskers extend from the edges of the box to show the range of the data. Outliers are plotted as separate dots.

**Figure 6:**
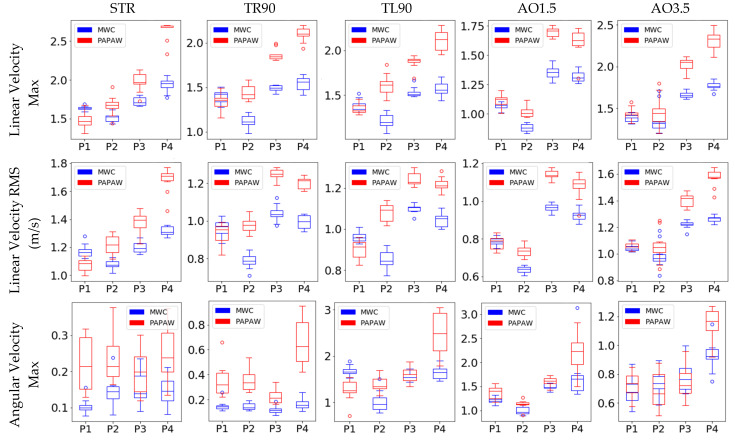

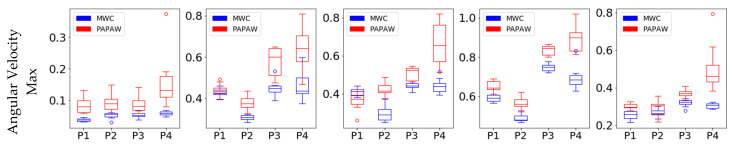
Summary of kinematic characteristics for all wheelchair maneuvers.

A summary of kinetic and kinematic data for all trials (a total of 10 for each participant) of all wheelchair maneuvers are shown in Figure 5 and Figure 6, respectively. These graphs display the differences between the MWC and PAPAW tests for all participants. Although the effects of PAPAW use on kinetic and kinematic measures varied across all maneuvers and between different participants, on average, participants applied less torque and travelled at a higher speed when using PAPAWs compared with manual wheels. For instance, the straightforward maneuvers for all participants had consistently higher RMS torque (both left and right wheels) for MWC tests compared with PAPAWs. When using PAPAWs, the RMS value of the wheelchair’s angular velocity during the straightforward maneuvers of all participants was higher compared with MWC tests.

Descriptive statistics of kinetic and kinematic measurements from MWC and PAPAW tests are reported in Table 5 and Table 6, respectively. Results of the Wilcoxon signed-rank tests comparing the kinetic/kinematic features between MWC and PAPAW tests are presented in Table 7. We found that three kinetic features (RMS torque of the left and right wheel, max torque of the right wheel) were significantly lower when using PAPAWs. All kinematic features, that are max/RMS of linear and angular velocity, were significantly higher for PAPAWs compared with MWC tests. The average number of pushes on each wheel was significantly reduced when using PAPAWs.

**Figure 7:**
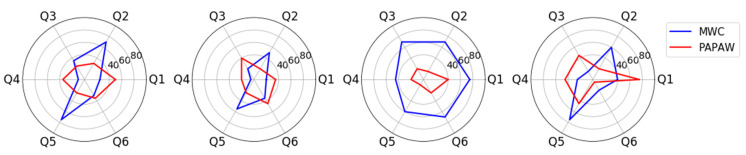
NASA-TLX scores for MWC and PAPAW tests. From left to right: Participants 1 to 4. Q1: Mental Demand’, Q2: ‘Physical Demand’, Q3: ‘Temporal Demand’, Q4: ‘Performance’, Q5: ‘Effort’, Q6: ‘Frustration’.

A comparison between the NASA-TLX scores of the MWC and PAPAW tests is presented in Figure 7. In general, the radar plot of PAPAW test (red lines) was smaller in area than MWC test (blue lines). The total score (i.e., the sum of all 6 domains) was lower when using PAPAWs (i.e., lower workload and more desirable) for all participants. A Wilcoxon signed-rank test showed that NASA-TLX scores of PAPAW tests were significantly lower than MWC tests (Z = −2.046, *p*-value = 0.041). All participants ranked the physical demand and effort of PAPAW propulsion lower than MWC tests. All participants, except Participant 3, perceived higher mental demand and lower performance when using PAPAWs compared with the MWC.

## 4. Discussion

To our knowledge, this is the first study to compare kinetic and kinematic characteristics of a MWC with and without PAPAWs for a variety of over-ground wheelchair maneuvers, including straight and turning motion. Previous experiments comparing MWCs and PAPAWs were performed mainly on a treadmill [20,27] or a dynamometer [17,19], which may not represent the wheeling characteristics during over-ground propulsion. Considering the differences between over-ground and dynamometer measurements [32], we believe the kinetic and kinematic measurements in our study can provide more realistic estimates of the performance benefits of PAPAWs. 

The outcomes of the MWC and PAPAW tests demonstrate that using PAPAWs can significantly reduce propulsion effort (i.e., RMS torque) and push frequency. For instance, our finding regarding the effects of PAPAW use on reducing propulsion effort (e.g., peak force on the pushrim) is in agreement with previous research, in which a significant reduction in the pushrim force was found when using PAPAWs compared with manual wheels [20,21].However, in contrast to our results, some previous studies did not find a significant difference in the propulsion frequency between MWCs and PAPAWs [20,26]. We believe that this could be related to the limitations of their experimental setup, in which all tests were performed on a dynamometer or a treadmill. Considering the risk factors associated with high propulsive forces/torques and push frequency [33], we speculate that PAPAWs may be effective in reducing or delaying the onset of upper extremity injuries.

Although all participants were instructed to wheel at a self-selected and comfortable speed, we found that the linear and angular velocities of the wheelchair were significantly higher when using PAPAWs. This is in line with findings of previous research, reporting an increase in the linear velocity during PAPAW propulsion [17,34]. The lower task completion time recorded in several maneuvers is due to the increased linear velocity when using PAPAWs. This feature can further contribute to improving the efficiency of wheelchair propulsion. By comparing the values of the angular velocity of the wheelchair for the straightforward maneuvers we concluded that participants had better control over the direction of motion when using the MWC compared with PAPAW tests. Potential reasons for this are that small variations of the side-to-side user input to the pushrims are magnified when using PAPAWs. Additionally, the side-to-side variations are more apparent when moving with higher linear velocities. Our data provided quantitative evidence supporting qualitative observations of previous studies regarding difficulties maneuvering PAPAWs [27,28].

The results of our subjective task load assessments were aligned with the quantitative kinetic and kinematic measurements. The significant reduction of the propulsion effort (i.e., RMS torque) when using PAPAWs was reflected in lower scores for physical demand and effort of PAPAW propulsion compared with manual wheels. Higher mental demand and frustration among some participants could be associated with less (precise) control over the wheelchair motions, as suggested previously [20]. The NASA-TLX scores of our skilled wheelchair user (Participant 3) for MWC tests were notably lower than the rest of the participants. One reason for this could be that the MWC tests were performed with a heavier and less maneuverable wheelchair than his personal wheelchair. 

The main limitation of this study is the small sample size, with the majority of participants being able-bodied individuals. Therefore, our findings may not be generalizable to other groups of wheelchair users. All study experiments were performed under controlled conditions (i.e., smooth level concrete) and did not include the normal variety of wheelchair activities performed daily and in different environments. Future studies should investigate other wheelchair maneuvers (e.g., moving up a ramp) and on different indoor/outdoor terrains. Despite these limitations, we believe that the findings of this study expanded the knowledge of how PAPAWs may improve propulsion efficiency when performing daily life wheelchair activities.

## 5. Conclusions

We performed quantitative and qualitative assessments of MWC and PAPAW propulsion when performing common daily life wheelchair maneuvers. Positive associations were found between using PAPAWs and exerting lower propulsion effort. Our subjective assessments revealed preliminary evidence in favour of using PAPAWs and supported our quantitative findings. The hardware setup developed in this work can provide a foundation for the future development of more advanced PAPAW controllers with a focus on improving overall wheelchair controllability and maneuverability.

## Figures and Tables

**Table 1:** Technical specification for the BCIT wheelchair.

Powered Wheels	
Motor (×2)	Grin All-axle, Direct-drive, 4 kg, KV: 7.5 rpm/V, KT: 1.2733 Nm/A,
Motor Controller	RoboteQ SBL2360T
Battery (×4)	LiGo: 36V DC, 2.7 A-hr, 0.61 kg
Maximum speed	10 km/h
Range	Greater than 12 km at 5 km/h on a single battery charge
Total Mass	Mass added to wheelchair is <15 kg
**Elevation^TM^ wheelchair**	
Seat	
Width	420 mm
Depth	430 mm
Backrest height	370 mm
Rear wheels	610 mm, one cross stainless-steel spokes, aluminum pushrims
Rear tires	Pneumatic, inflated to rated tire pressure of 90 psi
Front casters	Ø 100 mm, Polyurethane tires

**Table 2:** Electronic Components of the BCIT Wheelchair.

**Wheels**	
Microcontroller	Teensy 3.6
Bluetooth Module	HC-05
Battery	3.7 Volt Lithium Ion 2000 mAh
Hall effect sensor	HAL805 (TDK-Micronas, Freiburg, Germany)
IMU	MPU 6050 6-DoF Accelerometer and Gyro
**Frame**	
IMU	BNO055 (Bosch^®^, Reutlingen, Germany), Accelerometer and Gyro
Microcontroller	Teensy 4.1
Bluetooth Module	RN-42

**Table 3:** Demographic information of study participants.

No.	Gender	Age (Years)	Diagnosis	Weight (kg)	Years of Experience Using Manual Wheelchairs	Years of Experience Using PAPAWs
P1	F	33	AB ^1^	52	3	3
P2	M	30	AB	68	6	5
P3	M	51	SCI ^2^	68	32	2
P4	M	33	AB	75	4	4

^1^ Able-bodied; ^2^ spinal cord injury.

**Table 4:** Wheelchair maneuvers.

Maneuver	Description
Straight forward (STR)	Start from rest, move straight forward, stop 10 m away from the starting point.
Avoid obstacles (AO1.5, AO3.5)	Maneuver around obstacles that are placed 1.5 or 3.5 m apart.
Turn 90° (TL90, TR90)	Start from rest, move straight forward 5 m, turn left/right 90°, move straight forward, stop 5 m after the turn.
Turn in-place (TL180, TR180)	Turn 180° left/right in place.

**Table 5:** Descriptive statistics of kinetic measurements (N = 200).

Wheel Type	Statistics	Torque (Nm)				Push Frequency	
		Max		RMS			
		Left	Right	Left	Right	Left	Right
MWC	Mean ± std	13.70 ± 0.92	16.16 ± 0.79	6.44 ± 1.30	6.93 ± 1.32	6.9 ± 1.0	7.1 ± 1.2
	Median	13.74	16.32	6.40	6.92	7.0	7.0
	Range	[10.84, 17.47]	[12.26, 17.58]	[3.61, 9.68]	[4.24, 9.80]	[4.0, 10.0]	[5.0, 11.0]
PAPAW	Mean ± std	13.34 ± 1.77	14.16 ± 2.92	5.44 ± 1.51	5.67 ± 1.81	4.7 ± 1.2	4.5 ± 1.3
	Median	14.44	16.05	5.21	5.39	5.0	4.0
	Range	[9.60, 15.10]	[7.08, 16.97]	[2.77, 9.05]	[2.89, 9.79]	[3.0, 8.0]	[1.0, 9.0]

**Table 6:** Descriptive statistics of kinematic measurements (N = 200).

Wheel Type	Statistics	Linear Velocity (m/s)		Angular Velocity (rad/s)	
		Max	RMS	Max	rms
MWC	Mean ± std	1.44 ± 0.26	1.02 ± 0.17	0.78 ± 0.61	0.35 ± 0.20
	Median	1.45	1.02	0.76	0.37
	Range	[0.83, 2.06]	[0.60, 1.36]	[0.07, 3.13]	[0.03, 0.83]
PAPAW	Mean ± std	1.70 ± 0.41	1.15 ± 0.24	0.94 ± 0.69	0.44 ± 0.24
	Median	1.66	1.13	0.81	0.42
	Range	[0.97, 2.70]	[0.69, 1.77]	[0.12, 3.04]	[0.06, 1.02]

**Table 7:** Z-value Wilcoxon signed-rank tests (N = 200).

Torque (Nm)				Linear Velocity (m/s)		Angular Velocity (rad/s)		Push Frequency	
Max		RMS							
Left	Right	Left	Right	max	rms	max	rms	Left	Right
−1.35	−8.76 *	−10.84 *	−11.72 *	−10.38 *	−10.02 *	−7.72 *	−10.95 *	−11.65 *	−12.13 *

* *p*-value < 0.001.

## Data Availability

The data presented in this study are available on request from the corresponding author. The data are not publicly available due to privacy issues.
